# Prognostic information of serial plasma osteopontin measurement in radiotherapy of non-small-cell lung cancer

**DOI:** 10.1186/1471-2407-14-858

**Published:** 2014-11-21

**Authors:** Christian Ostheimer, Matthias Bache, Antje Güttler, Thomas Reese, Dirk Vordermark

**Affiliations:** Department of Radiation Oncology, Martin Luther University Halle-Wittenberg, Klinik und Poliklinik für Strahlentherapie, Martin Luther Universitaet Halle-Wittenberg, Ernst-Grube-Strasse 40, 06097 Halle (Saale), Germany

**Keywords:** Osteopontin, Radiotherapy, Non-small-cell lung cancer, Tumor hypoxia, Prognostic factors, Plasma biomarker

## Abstract

**Background:**

Circulating baseline levels of the plasma-protein osteopontin (OPN) have been suggested as a prognostic indicator in chemotherapy and surgery for lung cancer. However, the role of this hypoxia-related protein in radiotherapy of lung cancer is unclear. We previously demonstrated the prognostic effect of baseline OPN plasma levels which was increased by co-detection with other hypoxia-related proteins in the radical radiotherapy of non-small-cell lung cancer (NSCLC). This prospective clinical study investigated whether serial OPN measurements during and after curative-intent radiotherapy for NSCLC provide additional or superior prognostic information.

**Methods:**

Sixty-nine patients with inoperable NSCLC were prospectively enrolled (55 M0, 14 M1). OPN plasma levels were measured before (t0), at the end (t1) and four weeks after radiotherapy (t2) by ELISA, compared between M0 and M1 patients and correlated with clinicopathological parameters. OPN levels were monitored over time and correlated with prognosis in M0-stage patients treated by radical 66-Gy radiotherapy ± chemotherapy.

**Results:**

Pre-treatment OPN levels were associated with T stage (p = .03), lung function (p = .002), weight loss (p = .01), tumor volume (p = .02) and hemoglobin concentration (p = 04). M1 patients had significantly elevated OPN levels at all time points (p < .001). Patients with increasing OPN levels after radiotherapy had inferior freedom from relapse (p = .008), overall survival (p = .004) and disease-free survival (p = .001) compared to patients with stable or decreasing OPN levels. The risk of relapse in patients with increasing or stable OPN levels after radiotherapy was increased by a factor of 2.9 (p = .01). Patients with increasing post-treatment OPN levels had a 3.1-fold increased risk of death (p = .003). In an exploratory multivariate model, post-treatment OPN level changes but not absolute baseline OPN levels remained an independent prognostic factor for overall survival (p = .002) with a 3.6-fold increased risk of death, as well as N stage (p = .006).

**Conclusions:**

Our results suggest that OPN level changes over time, particularly post-treatment, may yield additional prognostic information in curative-intent radiotherapy of NSCLC.

## Background

Despite recent advances in chemo-radiotherapy of advanced non-small-cell lung cancer (NSCLC) [1], tumor hypoxia remains a critical and common feature of solid tumors which limits radiosensitivity and adversely impacts prognosis and response to radiotherapy [2]. In order to overcome hypoxic radiation resistance and improve prognosis after radiotherapy, feasible and efficient methods to predict clinically significant tumor hypoxia need to be identified [3–5].

So-called “endogenous hypoxia markers” have been suggested as a promising non-invasive approach to select patients with hypoxic tumors before radiotherapy for hypoxia-targeted therapies which are currently under investigation [6, 7].

The extra-cellular matrix protein osteopontin (OPN) is one of these hypoxia-related markers and of particular interest due to its potential association with tumor oxygenation which is prognostic in lung cancer. OPN plasma levels correlate with intra-tumoral pO_2_, measured by polarographic needle electrodes, in NSCLC [8]. Overexpression and elevated baseline (i.e. pre-therapeutic) plasma levels of OPN are associated with inferior prognosis in several human malignancies [9, 10].

The advantage of OPN lies in its easy detection in plasma or serum of cancer patients [11] and in its probable relation to hypoxic radiation resistance: In radiotherapy of head-and-neck cancer, pre-treatment OPN plasma levels were able to successfully predict tumor hypoxia and to identify patients who benefitted from treatment with the hypoxic radiosensitizer nimorazole [12, 13].

In chemo- [10] and surgical therapy of lung cancer [14], the prognostic relevance of pre-treatment OPN plasma levels has been documented: Compared to baseline OPN, plasma levels have been shown to decrease after resection of NSCLC. However, equivalent data for radiotherapy of NSCLC is still missing, particularly for serial OPN detection during and after radiotherapy.

We previously showed that baseline “hypoxic profile” of OPN in combination with other hypoxia-related proteins, namely carbonic anhydrase IX (CAIX) and vascular endothelial growth factor (VEGF), but not baseline OPN alone, was an independent predictor of survival on multivariate analysis [15].

The aim of this study was to evaluate whether serial OPN detection levels during and after curative-intent radiotherapy for NSCLC and assessing plasma level changes over time might provide superior prognostic information compared to baseline marker detection.

## Methods

### Study population

From November 2008 to June 2010, 69 patients with locally advanced inoperable NSCLC were prospectively enrolled at the Martin Luther University Halle-Wittenberg Dept. of Radiation Oncology. The protocol was approved by the Ethics Committee of the Medical Faculty, Martin Luther University Halle-Wittenberg. Inclusion criteria were age ≥18 years, histologically confirmed untreated NSCLC, indication for definitive radiotherapy as determined by interdisciplinary tumor board and signed informed consent. Staging was performed according to the TNM classification of malignant tumors (7th edition). Patients were grouped into two different cohorts depending on the presence or absence of distant metastases (M0 or M1 situation). Treatment consisted of curative-intent radiotherapy (2 Gy/day, 5 fx/week) to a total dose of 66 Gy or concomitant chemoradiation with cisplatin and and vinorelbine. M1 patients received at least 36 Gy of palliative-intent radiotherapy (3 Gy/day, 5 fx/week). Patients were followed up initially 4 to 6 weeks after radiotherapy and then observed at 3-monthly intervals.

### Plasma samples

Serial blood samples were obtained before (t0), at the end (t1) and 4 weeks after radiotherapy (t2) using EDTA. After centrifugation, plasma was removed and stored at −80°C. ELISA (Human Osteopontin Assay, IBL Ltd., Japan) was performed and optical density was measured in duplicate according to manufacturer’s instructions. OPN plasma concentration was determined using the standard curve supplied by the kit and is reported in ng/ml (± one standard deviation, SD).

### Statistical analysis

The median OPN plasma level was used as a cut-off value. Changes in OPN levels from one measuring time point to the other were divided into three categories (based on baseline OPN level, t0): increase by ≥10%, stability between −10% and +10% and decrease by 10%, as previously defined [16].

Non-parametric tests were used to test for differences in OPN levels between two groups and to determine association of pre-treatment (t0) OPN levels with patient, disease and treatment characteristics.

Pearson’s test evaluated the correlation between OPN plasma levels and Wilcoxon’s test compared pre-treatment with post-treatment OPN plasma levels.

Palliative (M1, n = 14) patients were only evaluated for comparison of patient characteristics and only curative-intent (M0-stage, n = 55) patients were considered for the endpoints overall survival (OS, from start of radiotherapy until death or last seen in follow-up), freedom from relapse (FFR, with death before relapse as a censoring variable) and disease-free survival (DFS, with relapse counting as an event and death before relapse as a censoring variable). These endpoints were analyzed by the Kaplan-Meier method and differences between survival curves were tested with the log-rank test. Univariate and multivariate Cox hazards regression analyses were performed to identify prognostic factors in an exploratory model for OS. The relative risk was evaluated with the х^2^-test and is reported with 95% confidence interval (CI).

All statistical analyses were performed using SPSS (version 18), statistical significance was accepted with two-sided p-values < .05.

## Results

### Demographics and patient characteristics

Clinical patient characteristics and demographics are displayed in Table 1. The entire group (n = 69) included 55 patients in M0 stage who were treated with curative intent and 14 patients in M1 stage treated with palliative intent. The association of pre-treatment OPN levels and patient characteristics was assessed in the entire patient cohort (n = 69) with pre-treatment OPN levels being split at the median (830.3 ng/ml). Mean follow-up time in living patients was 35 (18–48) months.

**Table 1:** **Patient, tumor and treatment characteristics of the curative-intent (M0) and palliative-intent (M1) patient cohort and the entire patient collective and the relationship of pre-treatment OPN levels (split at the median) with clinicopathological patient factors (p-value compares pre-treatment OPN levels higher and lower than the median (830.3 ng/ml) and refers to the entire patient collective (n=69)**

		All patients (n=69)		M0 patients (n=55)		M1 patients (n=14)		Pre-treatment OPN ≤ 830.3 ng/ml		Pre-treatment OPN > 830.3 ng/ml		
Characteristics		No.	%	No	%	No	%	No	%	No	%	*P*
Age	*mean years*	63.1		63.4		62		60.9		65.8		*.02*
Sex	*male*	61	(88)	47	(86)	14	(100)	30	(49)	31	(51)	*.48*
	*female*	8	(12)	8	(14)	0	(0)	5	(63)	3	(37)	
Hemoglobin	*mean g/dl*	12.1		12.1		12.1		12.5		9.3		*.04*
FeV1^1^	*mean %*	69		69		65		78.1		61		*.002*
weight loss	*yes*	22	(32)	12	(22)	10	(71)	7	(33)	15	(67)	*.01*
	*no*	47	(68)	43	(78)	4	(29)	28	(64)	16	(36)	
Histology	*SCC* ^2^	33	(48)	28	(51)	5	(36)	14	(42)	19	(58)	*.33*
	*Adeno*	29	(42)	24	(44)	5	(36)	11	(38)	18	(62)	
	*NOS* ^3^	7	(10)	3	(5)	4	(28)	3	(43)	4	(57)	
Differentiation	*well-moderate*	22	(31)	20	(36)	2	(14)	14	(64)	8	(36)	*.05*
	*poor*	30	(44)	25	(46)	5	(36)	16	(53)	14	(47)	
	*undifferentiated*	17	(25)	10	(18)	7	(50)	5	(29)	12	(71)	
UICC stage	*I-II*	3	(4)	3	(5)	0	(0)	3	(100)	0	(0)	*.33*
	*III*	52	(76)	52	(95)	0	(0)	27	(52)	25	(48)	
	*IV*	14	(20)	0	(0)	14	(100)	5	(36)	9	(64)	
T stage	*1-2*	25	(36)	20	(36)	5	(36)	17	(68)	8	(32)	*.03*
	*3-4*	44	(64)	35	(64)	9	(64)	18	(41)	26	(59)	
N-stage	*0-1*	10	(14)	8	(14)	2	(14)	4	(44)	5	(66)	*.5*
	*2-3*	59	(86)	47	(86)	12	(86)	30	(51)	29	(49)	
M stage	*0*	55	(80)	55	(100)	0	(0)	35	(64)	20	(36)	*.03*
	*1*	14	(20)	0	(0)	14	(100)	5	(36)	9	(64)	
Mean GTV^4^	*ml*	200.3		219.9		117.4		176.8		261.6		*.02*
	*not available*			0	(0)	1	(7)					
Radiotherapy		25	(36)	13	(24)	12	(86)					^*‡*^
Radio chemotherapy		44	(64)	42	(76)	2	(14)					
Mean total dose	*Gy*	57.9		62.9		38.2						^*‡*^
Mean single dose	*Gy*	2.2		2		2.9						
Mean follow-up	*months*											^*‡*^
	*all patients*	14.6		16.7		6.5						
	*living patients*	34.9		34.3		4.16						

^1^forced expiratory 1-second volume, % of normal value ^2^squamous-cell carcinoma ^3^not otherwise specified ^4^gross tumor volume ^‡^p-value not applicable (pre-treatment OPN was not evaluated for treatment parameters).

Patients with higher age generally had higher pre-treatment OPN levels (p = .02) and elevated OPN plasma levels before radiotherapy were found in patients with low hemoglobin (p = .04) and poor lung function (FeV1, forced expiratory volume ≤ median: 909.7 ng/ml vs. FeV1 > median: 690.3 ng/ml, p = .002). Significant weight loss (≥6% body weight in 6 months) was associated with elevated OPN levels (1002.7 vs. 726.1 ng/ml, p = .01). Patients with larger tumors (higher T stage) and gross tumor volume (GTV, ml) had higher OPN plasma levels before radiotherapy (T1-2 vs. T3-4: 688.9 vs. 859.3 ng/ml, p = .03; GTV ≤ vs. > median: 141.5 vs. 213.3 ng/ml, p = .02). M1 patients had significantly elevated median pre-treatment OPN levels compared to M0-stage patients (1279.6 vs 816.6 ng/ml, p < .0001).

### Baseline OPN levels, marker correlation and intratherapeutic changes

Median OPN plasma levels before radiotherapy (t0), at the end of radiotherapy (t1) and 4 weeks after radiotherapy (t2) and their changes are shown in Table 2.

**Table 2:** **Median OPN plasma levels (ng/ml±SD) before (t0), at the end (t1) and four weeks after radiotherapy (t2) in curative-intent, palliative-intent and all patient (p- values refer to difference between curative and palliative cohort)**

	Curative (M0)	N	Palliative (M1)	N	All (M0+M1)	N	*P*
t0	816.6 (±430)	55	1279.6 (±1125)	14	879.6 (±674.3)	69	*< .0001*
t1	789.3 (±373.8)	52	1068.7 (±1487)	12	811.1 (±787.7)	64	*< .0001*
t2	674.5 (±547.2)	43	1238 (±1529.4)	6	703.4 (±787.7)	49	*< .0001*
t0 to t1^*^	-6.4 (±62.4)	52	+6 (61.6)	12	-3.7 (±62)	64	*.979*
t1 to t2^*^	-5.2 (±55.7)	41	-19.9 (±94.1)	5	-5.2 (±59.6)	46	*.963*

^*^median relative changes in %.

A strong positive correlation between pre-treatment OPN levels and both end-of-treatment OPN plasma levels (t1, r = .62, p < .0001) and those measured four weeks after radiotherapy (t2, r = .5, p = .001) was seen. Plasma levels four weeks after treatment (t2) also correlated with OPN levels at the end of treatment (t1, r = .31, p = .03). At all three time points, OPN plasma levels of M1 patients were significantly higher than in M0 patients (p < .0001).

Both in the entire patient cohort and in the curative-intent subgroup, median OPN levels decreased during (t0 to t1) and after radiotherapy (t1 to t2). In palliative-intent patients, OPN plasma levels decreased during, but increased again after radiotherapy. However, these changes remained insignificant (p = .1).

### OPN and freedom from relapse in curative-intent (M0) patients

After a mean follow-up time of 35 (18–48) months for living patients, 69% of curative-intent patients had data on tumor recurrence and 66% of these patients had relapsed.

**Figure 1:**
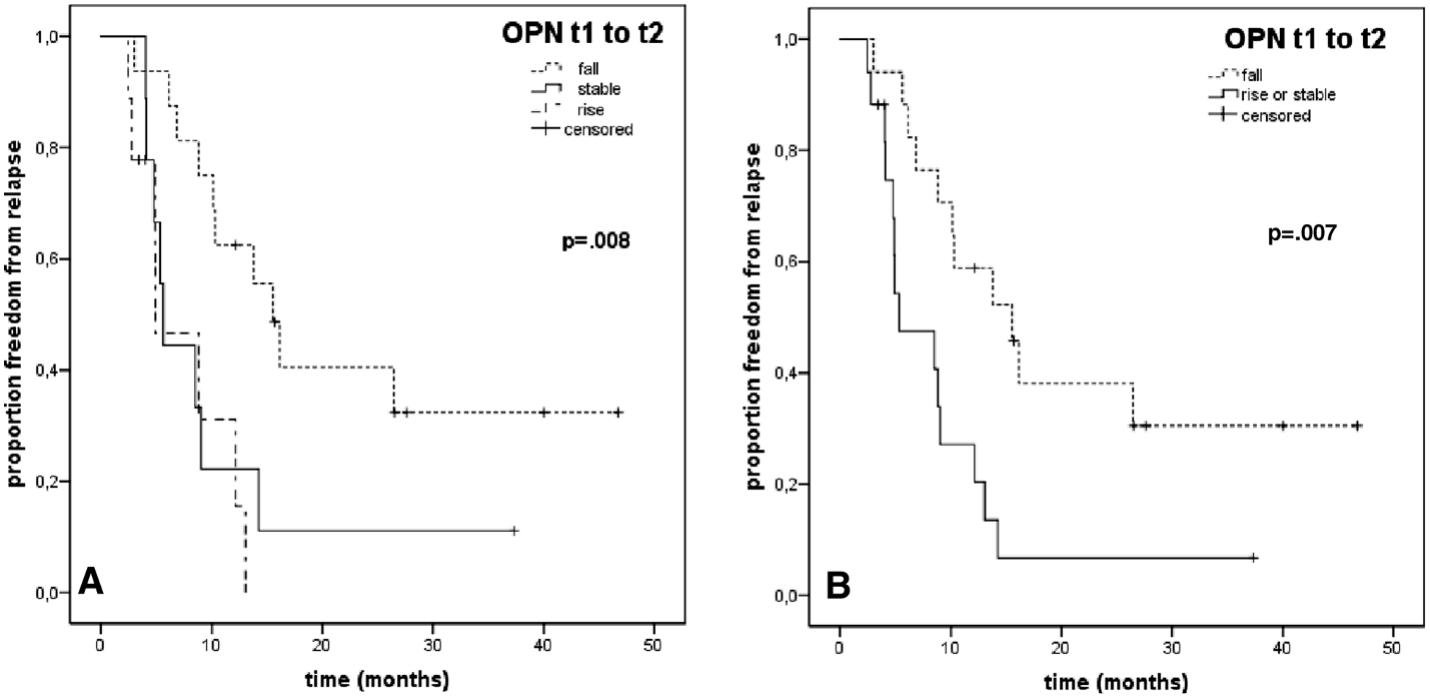
**Freedom from relapse in curative-intent (M0) patients in (A) increasing (rise** ≥ **+10%) vs. decreasing (fall** ≥ **−10%) vs. stable (between −10% fall and +10% rise) OPN levels after radiotherapy (t1 to t2) and (B) increasing or stable vs. decreasing posttreatment OPN levels in Kaplan-Meier analysis.**

Median FFR after radiotherapy is shown in Figure 1A and was 15.6 months (95%-CI [11.6-19.5]) in patients with decreasing (n = 18), 5.6 months (95%-CI [4.8-6.5]) in patients with stable (n = 9) and 4.9 months (95%-CI [.2-9.6]) in patients with increasing (n = 11) OPN levels after treatment (t1 to t2, p = .008). The risk of relapse was significantly elevated in patients whose OPN plasma levels were stable (rr = 2.8, 95%-CI [1.1-7.3], p = 0.03) or increased after treatment (rr = 4.2, 95%-CI [1.5-11.9], p = .006) compared to patients with decreasing post-therapeutic OPN levels.

When patients with similar FFR were grouped together (i.e. patients with increasing or stable vs. patients with decreasing post-treatment OPN levels), the effect on FFR was more pronounced: Median FFR in patients whose OPN levels were stable or increased after radiotherapy was 5.3 months (95%-CI [0.8-9.8]) as opposed to patients with decreasing post-therapeutic OPN levels who had a median FFR of 15.6 months (95%-CI [8.7-22.4], p = .007), Figure 1B. The relative risk for relapse was increased by a factor 2.9 (95%-CI [1.3-6.6]) for patients with increasing or stable OPN levels after radiotherapy (p = .01).

### OPN and overall survival in curative-intent (M0) patients

In curative-intent patients with complete follow-up data (n = 43), mean OS was 17.1 (.6-47.7) months. OPN plasma levels 4 weeks after treatment tended to be elevated in patients who later died during follow-up compared to patients who were still alive (711.9 ng/ ml vs. 563.5 ng/ml, p = .6). Absolute OPN plasma levels before but not at the end or after radiotherapy were associated with OS. Patients with elevated pre-treatment OPN levels (≥ median) had significantly reduced OS compared to those with low OPN plasma levels (< median, p = .03).

**Figure 2:**
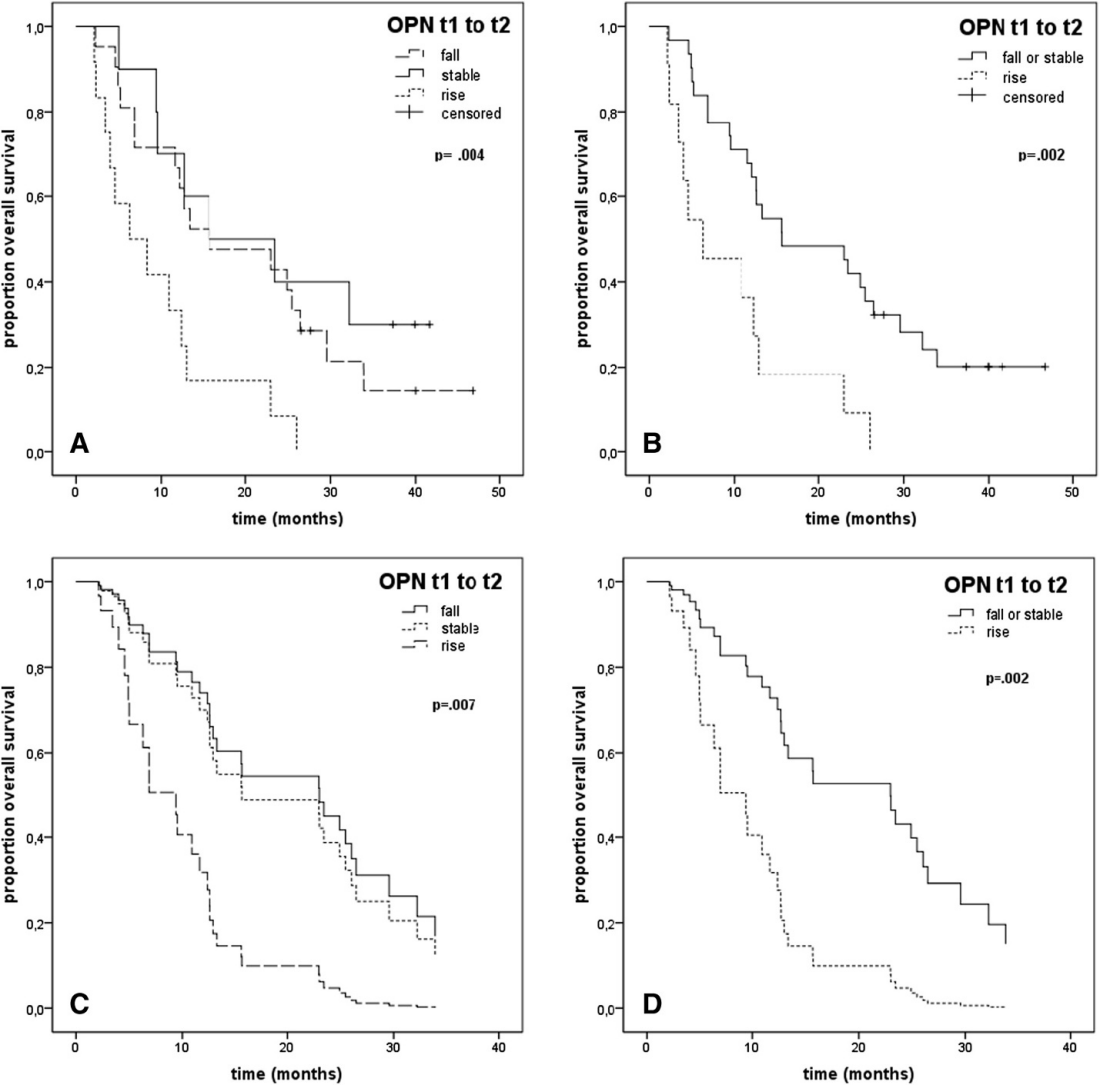
**Overall survival in curative-intent (M0) patients in Kaplan-Meier analysis (A and B) and Cox proportional hazards model (C and D). (A)** increasing vs. decreasing vs. stable and **(B)** increasing vs. decreasing post-treatment OPN levels (t1 to t2), **(C)** increasing vs. decreasing vs. stable and **(D)** increasing vs. stable or falling OPN levels after radiotherapy (t1 to t2).

Median OS was 15.7 months (95%-CI [.3-31.2]) in patients with decreasing (n = 21), 15.3 months (95%-CI [0–32.3]) in patients with stable (n = 10) and 6.3 months (95%-CI [0–12.7]) in patients with increasing (n = 12) OPN levels after radiotherapy (t1 to t2, p = .004), Figure 2A. Patients with rising OPN levels after treatment (n = 12) had a significantly increased risk of death at 2.8 (95%-CI [1.3-6.2]) compared to patients with decreasing (n = 21) or stable (n = 10) post-treatment OPN levels (p = .008).

After patients with comparable OS had been grouped together, we found that patients with post-therapeutically (t1 to t2) decreasing or stable OPN levels had a significantly (p = .002) superior survival (15.7 months, 95%-CI [4–27.4]) as opposed to patients with increasing OPN plasma levels after treatment (6.3 months, 95%-CI [0–13.7]). The latter patients also had an increased risk of death (3.1, 95%-CI [1.5-6.6], p = .003), Figure 2B.

### OPN and disease-free survival in curative-intent (M0) patients

**Figure 3:**
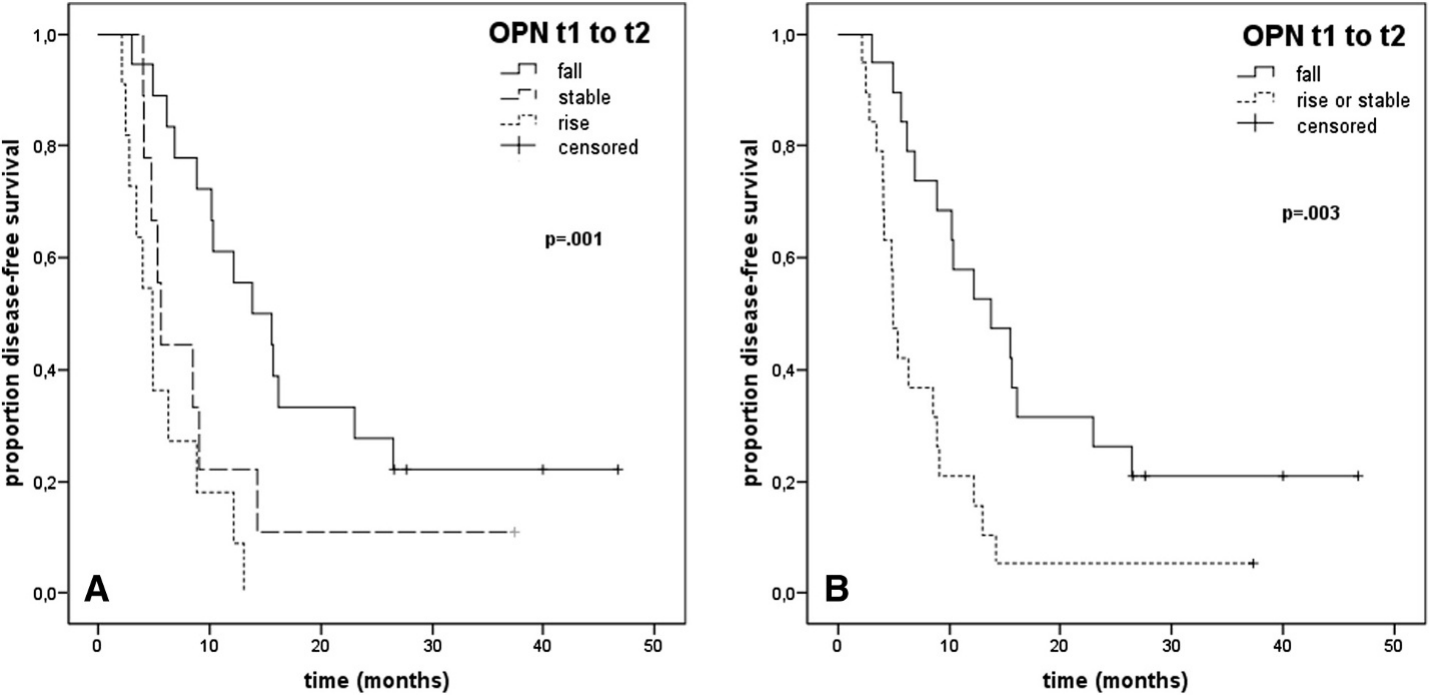
**Disease-free survival in curative-intent (M0 stage) patients in (A) increasing vs. decreasing vs. stable and in (B) increasing or stable vs. falling OPN levels after radiotherapy (t1 to t2) in Kaplan-Meier analysis.**

Mean DFS in 38 curative-intent patients with complete data on local or distant recurrence was 12.6 (.5-47.7) months. Absolute OPN plasma levels were not associated with DFS. In patients with decreasing OPN levels after treatment (t1 to t2, n = 18) mean DFS was 13.8 (6.8-20.9) months, in patients with stable post-treatment OPN levels (n = 9) DFS was 5.6 (4.8-6.5) months and it was 4.9 (3.3-6.4) months in patients with increasing post-therapeutic OPN values (n = 11, p = .001), Figure 3A. The relative risk of disease recurrence was significantly elevated in patients with stable (rr = 2.2, 95%-CI [1.1-5.4]) or increasing OPN levels from t1 to t2 time point (rr = 4.6, 95%-CI [1.9-11.1], p = .001) compared to patients with falling OPN levels after therapy. When patients with increasing or stable post-treatment OPN levels were compared to those with decreasing OPN levels from t1 to t2 measuring time point, DFS was significantly lower in the former group (4.9 [4.1-5.7] vs. 13.8 [6.3-21.3] months, p = .003), Figure 3B. Also, patients with increasing or stable OPN levels after therapy had a significantly increased risk to experience an event (rr = 2.9 95%-CI [1.4-5.8], p = .004).

### OPN in an exploratory predictive model for overall survival

An exploratory predictive model for OS, restricted to curative-intent (M0) patients with marker data at t0 and both t1 and t2 time points (n = 43), was constructed using a multivariate Cox proportional hazards model.

The baseline model included all categorical variables which were significantly associated with OS in the univariate analysis: gender, T stage, N stage, weight loss and age. In this model, only T stage (p = .02) and N stage (p = .01) were significant predictors of OS, weight loss displayed a trend (p = .07), age (p = .9) and gender (p = .4) were not significant. We then included post-treatment OPN level changes (t1 to t2) in the latter model as a categorical variable (rise vs. stable vs. fall) and found it was significant (p = .01) besides N and T stage. However, if absolute pre-treatment (t0) OPN plasma levels were included in the same baseline model (i.e. gender, T stage, N stage, weight loss, age), these were not significant (p = .6).

We then used a stepwise backward logistic regression in the baseline model including T stage, N stage, gender, weight loss, age and post-treatment OPN levels (t1 to t2). The final model (p = .01) consisted of OPN and N-stage, where limited lymphatic involvement (N0-1 vs. N2-3) had a reduced risk of death (rr = .1, 95% CI [.03-.5], p = .005). OS was lowest and the risk of death most considerably elevated in patients with increasing OPN levels after treatment (t1 to t2, rr = 3.8, 95%-CI [1.6-9]), followed by patients with stable post-treatment OPN levels (rr = 1.2, 95%-CI [.5-3]), p = .007, Figure 2C. If absolute baseline (t0) OPN plasma levels were added to the model, it was not significant (p = .3) and only N stage (p = .009) and relative OPN t1 to t2 plasma level changes (p = .007) remained significant in the final model.

When post-treatment OPN levels (t1 to t2) were compared, i.e. rising vs. falling or stable, they remained an independent prognostic factor for OS (besides N stage, p = .006) with a higher risk for death in patients with increasing OPN levels after radiotherapy (rr = 3.6, 95%-CI [1.6-8], p = .002), Figure 2D, Table 3.

**Table 3:** **Multivariate cox regression model for OS in curative-intent (M0-stage) patients (n=43)**

Variable	Comparison	Subject group^1^	Hazard ratio	95%-CI	*P*
**Gender**	Male vs. Female	*Male*	1.7	.7 - 4.9	.24
**T-stage**	T1-2 vs. T3-4	*T3-T4*	1.5	.3 - 7.3	.44
**Weight loss**	yes vs. no	*yes*	1.2	.4 - 3.5	.81
**N-stage**	N0-1 vs. N2-3	*N0-1*	.1	.03 - .5	*.006*
**OPN (t1 to t2)** ^**2**^	Decrease/Stable	*Increase*	3.6	1.6 - 8	*.002*

^1^focused variable category with significantly increased (gender, T-stage, weight loss, OPN) or decreased (N-stage) hazard ratio.

^2^relative OPN level changes from t1 to t2 measuring time point; increase (+10% rise), decrease (-10% fall), stable (between +10% rise and -10% fall).

## Discussion

In our study, pre-treatment OPN plasma levels were associated with advanced disease and tumor volume which is in agreement with the current literature [17–19]. Elevated OPN plasma levels before therapy were related to a poor oxygenation status of patients (i.e. poor lung function and low hemoglobin concentration) and paraneoplastic symptoms such as weight loss. This could be indicative of an association of OPN with an aggressive, biologically unfavorable and highly malignant hypoxic cancer phenotype [19–21]. We also found OPN plasma levels to be significantly higher in M1 patients at all measurement time points compared to M0 patients, suggesting that elevated pre-treatment OPN levels might reflect metastatic tumor burden [22] which is supported by the fact that in our study, M0 and M1 patients did not significantly differ in tumor size (T stage) or nodal involvement (N stage).

To the authors’ knowledge, this is the first study to evaluate the sequential detection of OPN plasma levels before, at the end of and four weeks after radical radiotherapy for NSCLC.

During radiotherapy, OPN levels remained mostly stable in palliative-intent (M1) patients which may be explained by their metastatic tumor load not being affected by radiation treatment [23, 24].

Both in the entire and curative-intent groups, overall OPN levels non-significantly decreased from before to after radiotherapy. This is consistent with the work of Snitcovsky et al. who reported similar pre- and post-treatment OPN plasma levels in patients with head-and-neck cancer undergoing radiochemotherapy [25]. In contrast, Blasberg et al. observed a significant decline of OPN plasma levels after resection of early stage NSCLC [14]. In our study however, patients were diagnosed with advanced-stage NSCLC and treatment was curative-intent radiotherapy. Assuming that the malignant tumor is the primary source of increased OPN plasma concentration, it is conceivable that an early decrease in OPN plasma levels may be observed after surgical removal of the tumor whereas with radiotherapy, tumoricidal effects are not as instant since tumor shrinkage occurs over the whole treatment course and tumor regression continues after the end of radiotherapy. This is supported by our finding that most prominent OPN level changes were observed after radiotherapy. This is in accordance with the results of Blasberg et al. who also noted the most obvious plasma level changes when OPN was evaluated after treatment [14].

The association of elevated OPN plasma levels with overall and progression-free survival was previously published for chemotherapy of head-and-neck cancer and NSCLC [10, 26, 27]. For curative radiotherapy of NSCLC, however, the prognostic relevance of serial OPN plasma level measurements has not been studied so far. We previously demonstrated the prognostic relevance of pre-therapeutic (baseline) OPN plasma levels as part of a “hypoxic profile” consisting of several markers [15].

In the present study, a homogeneous patient cohort (M0 patients) was included in survival analysis and prognostic information on multivariate analysis was seen only for the change of OPN from t1 to t2, but not absolute levels at any single timepoint.

Our data indicates that OS and DFS were superior in patients with decreasing OPN levels after radiotherapy. Prognosis and outcome was intermediate in patients with stable and it was lowest in patients with increasing OPN levels after treatment.

In surgically treated NSCLC, elevated OPN levels have been related to tumor recurrence [14]. In our study, FFR was inferior in patients with stable compared to patients with decreasing OPN levels after radiotherapy and FFR was lowest in patients with increasing post-radiotherapy OPN levels.

Notably, OS in our study was comparatively good in patients with stable or decreasing OPN levels, while it was significantly inferior in patients with increasing post-treatment OPN levels. In contrast, for FFR and DFS, only patients with decreasing OPN levels after radiotherapy had significantly superior outcomes while patients with stable or increasing post-treatment OPN levels had a poor FFR and DFS. One could speculate that for disease recurrence, stable post-treatment OPN levels might be indicative of residual or less responsive tumor after radiotherapy while increasing OPN levels might be related to a more resistant, progressive tumor, translating into reduced FFR and DFS. For OS however, decreasing or stable OPN levels might reflect locally controlled and stable distant disease after treatment, indicating a favorable prognosis while increasing OPN levels after treatment could be related to growth of initially present but occult micrometastases [28]. Given that survival in patients with advanced NSCLC is often determined by the development of distant metastases, monitoring of OPN plasma levels in the post-therapeutic window could provide additional prognostic information.

Since pre-treatment (baseline) OPN plasma levels have been proven to correlate with classic predictors of advanced disease including tumor size and volume, it has to be discussed whether OPN plasma levels merely reflect disease burden and whether the prognostic effect of OPN plasma levels and their changes might be an expression of tumor shrinkage. However, the multivariate analysis in this study demonstrates that OPN plasma levels and their changes, particularly in the post-treatment timeframe, remained significant predictors for OS independent from known prognostic factors including T stage which, in part, reflects tumor volume. Nevertheless, serial assessment of tumor shrinkage (by CT for example) at the time points of OPN readings could enhance uni- and multivariate analyses of further studies but were not available in the present study.

In this study, we hypothesized that serial detection of OPN plasma levels during and after radiotherapy might provide superior prognostic information compared to baseline OPN.

Multivariate analyses demonstrated that relative post-treatment OPN plasma level changes but not absolute pre-treatment OPN plasma levels were independent predictors of survival in the multivariate analysis. This is in accordance with our previous work where baseline OPN only was an independent predictor for survival if it was co-detected with other hypoxia-related proteins [15]. This supports the notion that the prognostic value of relative OPN plasma level changes after radiotherapy might be superior to that of absolute baseline OPN plasma levels detected before radiotherapy.

The current study furthermore supports findings of superior outcomes in NSCLC patients with low OPN levels before chemotherapy [26] and is in agreement with the findings of Dehing-Oberije et al. who reported OPN pre-treatment plasma levels not to be associated with OS in a multivariate prognostic model for inoperable NSCLC treated by combined chemoradiation or radiotherapy [29].

Certain limitations should be considered when evaluating the results of this hypothesis-generating study. Despite the homogeneity of the studied patient cohort, the relatively small patient number underlines the exploratory character of our work. Also, evaluation of our results in an independent data set would be desirable and since OPN may not be considered a direct surrogate of tumor hypoxia [30], a correlation with other surrogates of tumor oxygenation such as hypoxic (PET) imaging or other hypoxia markers would be useful [31, 32].

In future larger studies, patient subgroups with falling or rising OPN plasma levels during or after treatment could also be further classified by OPN velocity.

OPN plasma level detection in curative-intent radiotherapy of NSCLC might be of dual use: absolute pre-treatment OPN plasma levels, preferably in co-detection with other hypoxia-related proteins as part of a “hypoxic biomarker panel”, might help to identify patients with largely hypoxic, biologically aggressive and radioresistant tumors. These patients could be selected for individualized radiotherapy strategies which might be routinely available in future such as hypoxia modification or escalated radiotherapy in order to improve prognosis [15].

Monitoring relative OPN plasma levels changes during or especially after radiotherapy could provide additional prognostic information which might be potentially useful in the identification of patients with high risk of death and relapse after radiotherapy. This could be beneficial in patient stratification and the decision-making process for post-radiotherapy treatment concepts.

Patient selection strategies for treatment individualization and modification could be further amended by other dynamic approaches such as PET-CT or hypoxia-specific PET imaging during radiotherapy which enable earlier response detection [31, 33].

## Conclusion

In conclusion, our study generated first evidence that particularly post-treatment changes of OPN plasma levels may be predictive for FFR, OS and DFS which supports the further evaluation of serial detection of OPN plasma levels in the curative-intent radiotherapy of locally advanced NSCLC [34].

## Abbreviations

OPN: Osteopontin
OS: Overall survival
FFR: Freedom-from-relapse
DFS: Disease-free survival
NSCLC: Non-small-cell lung cancer
ELISA: Enzyme-linked immunosorbent assay
EDTA: Ethylenediaminetetraacetic acid.
